# Comparison of Hypomethylator Monotherapy with Hypomethylator plus Chemotherapy for Intermediate/High-Risk MDS or AML: A Meta-Analysis

**DOI:** 10.7150/jca.40614

**Published:** 2020-03-04

**Authors:** Jiang Ji, Miao Chen, Bing Han

**Affiliations:** Department of Hematology, Peking Union Medical College Hospital (PUMCH), Chinese Academy of Medical Sciences and Peking Union Medical College, Beijing, 100730, China

**Keywords:** myelodysplastic syndromes, acute myeloid leukemia, hypomethylating agent, monotherapy, HMA and chemotherapy combination therapy

## Abstract

**Aim**: This meta-analysis aimed to compare the efficacy, survival benefit and safety of hypomethylating agents (HMA) monotherapy and combination with chemotherapy in patients with intermediate/high-risk MDS or AML.

**Methods**: Related articles published between January 2009 and April 2019 were selected and patients were separated as monotherapy group and combination group for meta-analysis. Studies on HMA combination therapy were further divided into two subgroups according to the intensity of combined chemotherapy. Meanwhile, subgroups with similar patients' baseline characteristics were selected for further analysis. Complete response (CR) rate, overall response (ORR) rate, two-year overall survival (OS) rate, one-month and 24-month death rate and the proportion of adverse events (AE) were pooled and compared.

**Results**: 21 RCT or cohort studies with 1764 patients (1266 patients for monotherapy group and 498 patients for HMA combination group) were selected for meta-analysis. For the pooled data, the age of patients was significantly younger and the percentage of patients with favorable/intermediate cytogenetic risk was significantly higher in the HMA combination group than that in the HMA monotherapy group. Combination therapy group had a significantly higher CR and ORR rate (55% *vs* 22%, *P*=0.000 for CR and 67% *vs* 42%, *P*=0.000 for ORR), and a higher two-year OS rate (37% *vs* 21%, *P*=0.000). However, the incidence of infection and gastrointestinal disorder was significantly higher (51% vs 23% for infection, *P*=0.000; 21% vs 0% for gastrointestinal disorder, *P*=0.000) in combination group. In subgroups with different intensity of combined chemotherapy, all baseline characteristics were compatible except that the percentage of patients with favorable/intermediate cytogenetic risk was significantly lower (63% vs 88%, *P*=0.000) in the HMA + high-intensity chemotherapy subgroup, and this group presented with a lower CR and ORR rate (46% *vs* 65% for CR, *P*=0.000; 57% *vs* 79% for ORR, *P*=0.000), but a compatible two-month to 24-month death rate compared with HMA + low-intensity chemotherapy subgroup (9% *vs* 14% for 2-month death rate, *P*=0.060; 58% *vs* 65% for 24-month death rate, *P*=0.242). In subgroup with similar patients' baseline characteristics, 208 and 205 patients were included in combination group and HMA monotherapy group, respectively. Although combination group had a significantly higher CR rate (62% *vs* 24%, *P*=0.000) and ORR rate (68% *vs* 48%, *P*=0.000), it finally had a lower two-year OS (30% *vs* 45%, *P*=0.001) compared with monotherapy group, and the death rate was significantly higher since the ninth month in combination therapy group than that in the monotherapy group (42% *vs* 31%, *P=*0.032). In this subgroup, patients with HMA+ high-intensity chemotherapy had a compatible CR, ORR and 1.5-year OS rate as compared with baseline-compatible patients with HMA + low-intensity chemotherapy.

**Conclusions**: HMA combined with chemotherapy could increase CR rate and ORR rate in all patients. HMA combined with high-intensity chemotherapy can rescue the 2-year OS with less favorable cytogenetic stratification to some extent. For patients with similar older age and risk stratification, combination therapy even had a lower long-term OS regardless of the intensity of combined chemotherapy.

## Introduction

Myelodysplastic syndromes (MDS) are acquired bone marrow disorders characterized by ineffective clonal hematopoiesis and “dysplastic” cell morphology 1. MDS presents with heterogeneity and the potential to evolve into acute myeloid leukemia (AML)^2^. Epigenetic changes including abnormal DNA methylation were proved to contribute to the onset of MDS and AML^3^, and hypomethylator, or so called hypomethylating agents (HMA) decitabine and azacitidine have become the first-line therapy for intermediate/higher risk MDS and AML patients unsuitable for hematopoietic cell transplantation (HSCT)^4^, ^5^. The reported complete response (CR) rate of HMA monotherapy on MDS and AML were limited to 10-40%, and the overall response (ORR) rate was limited to 40-70%^6^-^9^, and combination of HMA and other treatments were investigated to achieve a better outcome.

HMA in combination with chemotherapy like DA (daunorubicin + cytarabine), IA (idarubicin + cytarabine)^10^, CAG (cytarabine + aclarubicin + granulocyte colony stimulating factor (G-CSF)), HAA (homoharringtonine + aclarubicin + cytarabine), etc. are easy to obtain with relatively low price. *In vitro* study proved that HMA had a synergistic anti-leukemia effect with anthracycline^11^, and was likely to enhance the intensity of chemotherapy. Some retrospective studies reported a better CR rate and short term OS benefit for the combination therapy as compared with monotherapy with HMA^12^, ^13^. However, the outcomes reported from different centers are highly variable with relative small patients' number individually. Moreover, very few studies had compared HMA monotherapy and HMA combined with chemotherapy head to head. This meta-analysis aimed to compare the efficacy, survival benefit and safety of HMA monotherapy and combination therapy (with chemotherapy) in patients with intermediate/high-risk MDS or AML.

## Methods

### Study design and inclusion criteria

Databases of Pubmed, Embase, Pubmed Central and Web of Science were selected for study searching. English articles published between January 2009 and April 2019, with “MDS”, “myelodysplastic syndrome”, “myelodysplastic syndromes”, “AML”, “acute myeloid leukemia” or “acute myeloid leukaemia” in titles, and “decitabine” or “azacitidine” in titles or abstracts were screened. Keywords for regimens of chemotherapy were not included in study searching because regimens of chemotherapy were diverse and could be expressed in various ways. Studies neither on HMA monotherapy nor HMA combining with intensive therapy were excluded after reviewing the title, abstract, or full-text of the study.

Inclusion criteria of the meta-analysis were: 1) randomized controlled trials or cohort studies; 2) data were extractable and the classification of MDS or AML was based on WHO-2008 criteria^14^; 3) for articles with both newly diagnosed and refractory/ relapsed MDS or AML patients, data from each cohort were available; 4) therapies with HMA - decitabine or azacitidine alone, or HMA combined with chemotherapy; 5) with integrated information, including CR rate, ORR rate, and overall survival analysis like Kaplan-Meier survival curve. Articles on MDS with low risk IPSS, therapy-related MDS/AML (t-MDS/ AML) patients were excluded.

### Data extraction

The following data were extracted from the studies: 1) study information (first author, publication year, study type); 2) patient baseline characteristics (patients' number, age, gender, disease type based on WHO-2008 criteria, cytogenetic risk, Eastern Cooperative Oncology Group Performance State (ECOG PS) and gene mutations if possible); 3) therapy regimen; 4) median follow-up; 5) outcomes of HMA or HMA combination therapy (CR rate, ORR rate, two-year OS rate, one-month to 24-month death rate, record of adverse events (AE) and cause of death). Only data from the first-line therapy intervention group was extracted.

CR and ORR referred to patients' response state at the last cycle of induction therapy. The definition of ORR included CR, partial response (PR), marrow CR and hematological improvement for MDS, and CR and PR for AML. Two-year OS rate and death rate was extracted directly from the text, or from the Kaplan-Meier survival curve via the software Engauge Digitizer (Windows version 11.2)^15^.

Data from patients with HMA monotherapy or HMA in combination with chemotherapy were compared.

### Statistical analysis

Heterogeneity among studies was examined by *I*^2^ measure of inconsistency, and *I*^2^ >50% was considered as an existence of significant heterogeneity. Pooled data for patient baseline characteristics (age, male proportion and cytogenetic risk proportion) and final results were obtained via a fixed-effects model, or a random-effects model if a significant heterogeneity was observed. For the study with a high proportion of patients who had received HSCT after either HMA monotherapy or HMA combination therapy, the OS of patients were censored right before HSCT, or the study was deleted for OS meta-analysis. Patients' age was pooled from mean age and standard deviation, which was estimated from the reported median age and age range (or interquartile range), as the original data of each study was not available. All included studies on combination therapy were further divided into two subgroups according to the intensity of combined chemotherapy. As no head-to-head study comparing HMA monotherapy and HMA combination therapy met the inclusion criteria, to eliminate the difference of patient baseline characteristics that possibly existed between two therapy groups, subgroups with compatible age distribution and cytogenetic risk degree at the baseline were further selected and analyzed. Studies with compatible patient baseline characteristics in high or low-intensity of chemotherapy + HMA subgroups were also selected and analyzed. Data were pooled and estimated by software Stata (Windows version 13.0, StataCorp LP, College Station, Texas, USA) and compared via χ^2^ test by the software IBM SPSS Statistics (Windows version 25)^16^.

## Results

### Study selection

**Figure 1:**
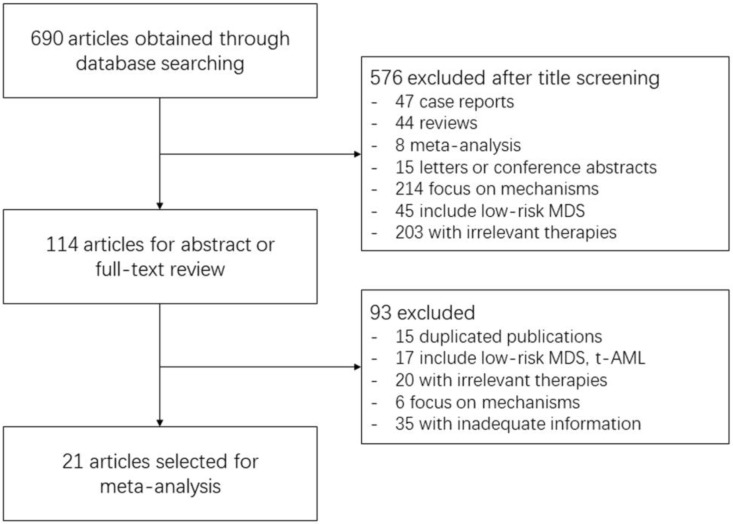
**Process of Study Selection.** The number of included or excluded articles in each screening step and the reasons of exclusion are demonstrated in the flow chart.

690 records were identified through database searching. After title screening, 576 articles were excluded mainly because of irrelevant direction like low-risk MDS, irrelevant therapies and molecular mechanism, and articles of case report, literature review, meta-analysis or conference abstracts. Another 93 articles were further excluded after reviewing the abstract or full-text. The majority of them were duplicated publications, studies involving low-risk MDS or t-AML patients, or with inadequate information. Some studies on irrelevant therapies were not discovered during title screening, and were excluded at this step. Finally, only 21 studies were included for meta-analysis. The detailed process of study selection is displayed in Figure 1.

For the combination subgroup analysis, Studies using standard dose of intensive chemotherapy (DA, IA, etc.) 17-20 were classified as HMA + high-intensity subgroup, while those using reduced dose of intensive chemotherapy 21-23 were taken as HMA + low-intensity subgroup. Studies with compatible age and cytogenetic risk were first selected according to the median age. As the median age of studies in combination therapy group ranged between 54.5 to 70, and that in HMA monotherapy was between 60 to 78, studies were narrowed down to those of median age between 60 to 70. Among them, studies of compatible age and proportion of favorable/intermediate cytogenetic risk were further screened, and six studies were selected as candidates. After excluding one study^24^ in monotherapy group with a high proportion of patients receiving HSCT and a consequent deviation in OS, five studies (Müller-Tidow et al., Li et al. and Krug et al.'s study on combination therapy^19^, ^20^, ^23^, and van der Helm et al. and Fenaux et al.'s study on monotherapy^9^, ^25^) were selected for subgroup analysis, and adding any other study to either subgroup would lead to a significant difference in either pooled estimate of age or proportion of cytogenetic risk. Same strategy was used for selecting studies with compatible baseline characteristics in high or low-intensity of chemotherapy + HMA subgroups, and only Krug et al. and Li et al.'s study^20^, ^23^ were selected and analyzed.

### Patients' Characteristics

Characteristics of the included patients in each study were presented in Table 1 and Table 2. 1764 patients in 21 studies were included in the meta-analysis, with 498 patients receiving combination therapy and 1266 receiving HMA monotherapy (decitabine or azacitidine alone). The induction therapies in combination therapy group included HMA in combination with CAG, or low-dose IA, or low-dose AA (aclacinomycin + cytarabine), or DA, and consolidation therapies included a combination of HMA and CAG, or a combination of HMA and cytarabine (Table 1). In monotherapy group, patients were treated with HMA alone as long as they can tolerate and had no disease progression. Most studies enrolled patients with ECOG PS of 0-2, but some lacked the detail information of ECOG distribution (Table 2).

The pooled male proportion of patients with combination therapy and HMA monotherapy group were 58% (95%CI: 54%-63%) and 65% (95%CI: 60%-70%) respectively (*P*=0.006). The pooled percentage of patients with favorable/intermediate cytogenetic risk was significantly higher in the HMA combination group (75%, 95%CI: 63%-86%) than that in the HMA monotherapy group (70%, 95%CI: 65%-74%, *P=*0.021), and patients were significantly younger in the HMA combination therapy group (estimated mean age of 63.9±11.8 year-old) than those in the HMA monotherapy group (70.6±9.5 year-old, *P=*0.000).

For subgroups with different intensity of combined chemotherapy, there were 315 patients in HMA + high-intensity chemotherapy group, and 183 patients in HMA + low-intensity chemotherapy group. There was no significant difference in patient's age (64.2±10.2 year-old and 63.5±10.3 year-old, *P*=0.464) and male proportion (57%, 95%CI: 51%-62%, and 62%, 95%CI: 55%-69%, *P*=0.225) between two subgroups. The proportion of favorable/intermediate cytogenetic risk was 63% (95%CI: 57%-68%) for HMA plus high-intensity chemotherapy group and 88% (95%CI: 80%-96%) for HMA plus low-intensity chemotherapy group (*P*=0.000).

For subgroups with compatible patient baseline characteristics, there were 208 patients in combination group and 205 patients in monotherapy group, respectively. There was no significant difference in patients' age (68.9±4.5 and 69.2±6.5 years old, *P=*0.587) and proportion of favorable/intermediate cytogenetic risk (73%, 95%CI: 59%-88%, and 70%, 95%CI: 64%-77%, *P*=0.523) between the two groups, but a significantly higher proportion of male in HMA monotherapy group (59%, 95%CI: 52%-66%, and 73%, 95%CI: 67%-79%, *P*=0.003). Among this group, Krug et al. and Li et al.'s study presented with compatible patient age (69.1±3.5 and 68±4.5 year-old, *P*=0.318), cytogenetic risk stratification (75%, 95%CI: 51%-99% *vs* 82%, 95%CI: 74%-90%, *P*=0.821) and male proportion (50%, 95%CI: 22%-78% *vs* 62%, 95%CI: 52%-72%, *P*=0.650).

### Publication bias

Publication bias was detected via the Begg test and the Egger test for the pool results for each group and no bias was found.

### Efficacy

The CR rate and ORR rate reported in each study were displayed in Table S1. A random model was used for pooling CR rate and ORR rate due to the high heterogeneity detected by *I^2^* test. The pooled estimate for CR rate was 55% (95%CI: 43%-68%) for patients with combination therapy, significantly higher than that for HMA monotherapy (22%, 95%CI: 18%-26%, *P*=0.000). The pooled estimate for ORR rate was 67% (95%CI: 49%-84%) and 42% (95%CI: 35%-48%) for patients with combination therapy and HMA monotherapy, respectively (*P*=0.000).

### Survival

Two-year overall survival (OS) rate was extracted for meta-analysis. Three studies were excluded for OS analysis because of a high proportion of patients receiving HSCT within two year after the therapy 17, 18, 24. A random model was used due to the high heterogeneity of the data detected by *I^2^* test and finally, the two-year OS rate was 37% (95%CI: 23%-51%) and 21% (95%CI: 13%-29%) for patients who had received combination therapy and HMA monotherapy, respectively (*P*=0.000, Table S1).

**Figure 2:**
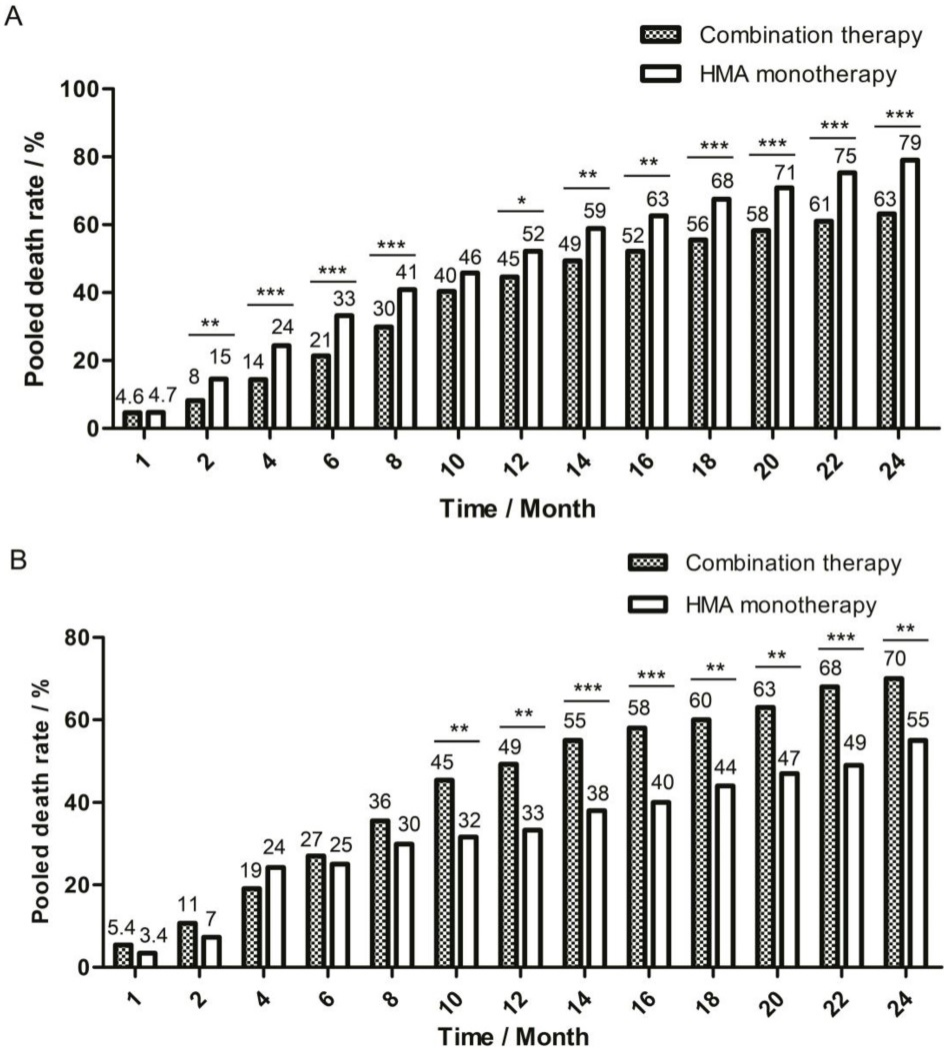
** One-month to 24-month pooled death rate of two therapy groups.** The death rate in each month was pooled and compared between combination therapy group and HMA monotherapy group. The bar of pooled death rate was displayed every two months after the first two months of follow-up. Pooled death rate was labeled on the top of each bar. (A) Pooled death rate of all enrolled studies in meta-analysis. (B) Pooled death rate of studies in age-compatible subgroup analysis. * *P*<0.05; ** *P*<0.01; *** *P*<0.001.

A fixed model was used for pooling one-month death rate, and a random model was used for the 2-month to 24-month death rate due to the different level of heterogeneity detected by *I^2^* test. The pooled death rate of combination therapy group was generally lower, but there were some time points during follow-up when there was no significant difference between the two therapy groups: in the first month, the death rate was 4.6% (95%CI: 2.0%-7.2%) for combination group and 4.7% (95%CI: 3.3%-6.6%, *P*=0.986) for monotherapy group and in the tenth month, the death rate was 40% (95%CI: 32%-49%) for combination group and 46% (95%CI: 37%-54%, *P*=0.090) for monotherapy group (Figure 2A).

### Safety

No detailed data of the adverse effects or cause of death for each month after therapy were available, and most studies recorded causes of death in the first 30 to 90 days of treatment. The major causes of early death were severe infection, cardiac disorders, hepatorenal syndrome, intracranial hemorrhage and gastrointestinal disorders for combination therapy group and disease progression, intracranial hemorrhage, pneumonia, sepsis and cardiac disorder for monotherapy group.

Studies that did not report AE or only reported AE of all grades were excluded for further analysis. AE were recorded during the induction therapy, and most studies reported the rate of grade 3-4 hematologic toxicities like severe neutropenia, thrombocytepenia, febrile neutropenia and anemia. The rate of grade 3-4 hematologic toxicities ranged from 50% to 95% for most studies, but less than 40% for some in HMA monotherapy group 26-29. A random model was used for pooling Common Terminology Criteria for Adverse Events (CTCAE) grade 3-4 infection, bleeding, cardiac disorder and gastrointestinal AE rate, and showed that a significantly higher infection rate (51%, 95%CI: 18%-85%) for combination therapy as compared with monotherapy group (23%, 95%CI: 12%-33% , *P=*0.000, Table S1). A significantly higher grade 3-4 gastrointestinal AE rate (21%, 95%CI: 10%-33% and 0, *P*=0.000, Table S1) was also found for combination therapy group. No significant difference was found in grade 3-4 bleeding rate (16%, 95%CI: 0%-37% and 8%, 95%CI: 2%-13%, *P*=0.084) and grade 3-4 cardiac disorder rate (5%, 95%CI: 0%-10% and 0, *P*=0.053, Table S1) between combination therapy group and monotherapy group. Other common non-hematologic AE included general deterioration for combination therapy group, and general deterioration, renal failure, hepatic dysfunction, hypokalemia for monotherapy group. The rate of grade 3-4 non-hematologic AE ranged between 2.5% to 12.6%.

### Subgroup analysis

For subgroups with different combined chemotherapy intensity, the pooled CR rate was 46% (95%CI: 35%-58%) and 65% (95%CI: 50%-80%, *P*=0.000), and the pooled ORR rate was 57% (95%CI: 37%-78%) and 79% (95%CI: 73%-85%, *P*=0.000) for HMA + high-intensity and HMA + low-intensity chemotherapy subgroup, respectively. However, no significant difference was found in two-year OS rate (42%, 95%CI: 32%-52% for high-intensity, 35%, 95%CI: 17%-54% for low-intensity, *P*=0.242), or the 2-month (14%, 95%CI: 5%-22% *vs* 9.4%, 95%CI: 9.0%-9.7%, *P*=0.060) to 23-month (58%, 95%CI: 42%-62% *vs* 64%, 95%CI: 48%-79%, *P*=0.374) death rate, while the one-month death rate was higher for high-intensity (12%, 95%CI: 5%-29% *vs* 4%, 95%CI: 0%-7%, *P*=0.008). The pooled CTCAE grade 3-4 infection rate was 66% (95%CI: 59%-73%) and 43% (95%CI: 0%-95%, *P*=0.000), and the pooled grade 3-4 cardiac rate was 12% (95%CI: 7%-16%) and 1% (95%CI: 0%-3%, *P*=0.000) for high and low-intensity subgroup, respectively. There was one study in each subgroup reporting grade 3-4 bleeding rate, which was 6% in high-intensity subgroup 17 and 28% for low-intensity subgroup 22.

For subgroups with compatible age and risk degree, the combination therapy group had higher pooled CR rate (62%, 95%CI: 40%-85% *vs* 24%, 95%CI: 7%-40%, *P=*0.000) and pooled ORR rate (68% , 95%CI: 48%-87% *vs* 48%, 95%CI: 41%-55%, *P=*0.000). However, the combination therapy group had much lower two-year OS rate (30%, 95%CI: 8%-53%* vs* 45%, 95%CI: 31%-60%, *P=*0.001) as compared with the monotherapy group. Different from the pooled data, the death rate of two groups did not show significant difference at the beginning of follow-up, but the death rate of combination therapy group increased faster than that of monotherapy group, and a significantly higher death rate was observed since the ninth month for the combination therapy group (42%, 95%CI: 37%-51% *vs* 31%, 95%CI: 14%-48%, *P=*0.032, Figure 2B). Though studies in this subgroup lacked the records of grade 3-4 AE like infection and bleeding, Li et al.'s study 23 in combination therapy group and van der Helm et al.'s study^25^ in monotherapy group reported causes of death : 16.5% of patients died of infection alone in combination group, while 0% patients in monotherapy group. For the baseline-compatible studies between high and low-intensity chemotherapy + HMA subgroup, no significant difference was detected in the CR rate (58%, 95%CI: 30%-86% *vs* 78%, 95%CI: 69%-87%, *P*=0.255), ORR rate (58%, 95%CI: 30%-86% *vs* 82%, 95%CI: 74%-91%, *P*=0.118), and 1.5-year OS rate (32%, 95%CI: 5%-58% *vs* 34%, 95%CI: 24%-44%, *P*=0.992) between Krug et al.'s study and Li et al.'s study. The CTCAE grade 3-4 cardiac disorder rate was 8% (95%CI: 0%-24%) for Krug's et al.'s study and 1% (95%CI: 0%-3%) for Li et al.'s study (*P*=0.552).

## Discussion

HMA (including azacitidine and decitabine) has been widely used for intermediate / high-risk MDS or AML unfit for HSCT or as the bridge therapy before HSCT. Due to the limited CR and ORR, combination therapy with different agents including combination chemotherapy had been investigated for the past several years. It had been shown in small patients cohorts that HMA in combined with chemotherapy improved the react rate^30^ and therefore, may benefit the final outcome. Although some new target medicine like BCL-2 inhibitor, PD-1 antibody, IDH1 inhibitor etc. have achieved great progress when combined with HMA for those diseases, most of them are still under clinical trials and not available for clinical practice. In this meta-analysis, publications for HMA or HMA combined with chemotherapy in which data were extractable were collected and analyzed to elucidate the possible value of combination with chemotherapy. As far as we know, it is the largest meta-analysis in this field so far, and more importantly, the first one involving survival analysis.

For the eligible pooled data, although other clinical characters before treatment were compatible, we found that patients in combination group had younger age and higher proportion of favorable/ intermediate cytogenetic risk than those in the monotherapy group. It is reasonable that doctors would give combination therapy for those with younger age and better general condition who were supposed to have a better tolerance and outcome. Under this circumstance, the combination therapy group achieved increased CR rate, ORR rate, and two-year OS rate. However, detailed analysis on death rate showed that the superiority in OS for combination therapy group was not constant and disappeared at some time points during follow-up. Comparison on AE also indicated that CTCAE grade 3-4 infection and gastrointestinal AE rates were both significantly higher in combination therapy group. Since chemotherapy was continuously given during the follow-up period, the toxicity might accumulate and impair the survival advantage in the end.

Interestingly, comparison of subgroups with high or low -intensity of chemotherapy + HMA showed that although a lower CR rate and ORR rate was detected in the high intensity group, probably due to the higher unfavorable cytogenetic risk percentage at the beginning, the two-year OS rate was similar between the two subgroups, indicating that in this relatively younger patient group (63∼64 year- old) a higher intensity of the combined chemotherapy could overcome the negative effect of unfavorable cytogenetic risk and benefit the survival. However, Further comparison of baseline-compatible studies with high or low intensity chemotherapy + HMA indicated that for older patients (nearly 70 year-old), there was no significant difference between the two subgroups in treatment efficacy and long-term OS.

To further exclude the influence of age and cytogenetic risk, patients from different treatments who had compatible age and cytogenetic risk were analyzed. For this sub-cohort, the two treatment groups had very similar age of nearly 70 year-old (68.9±4.5 and 69.2±6.5 years old, respectively) and similar risk grade. As expected, although the combination therapy had a higher CR and ORR rate, it came with poorer two-year OS. Detailed comparison even showed that although not significant, death rate seemed to be higher since the first month after therapy, and became significantly higher since the ninth month after therapy in combination group. Even in the early time after therapy, higher CR and ORR rate did not improve OS, and this may be explained by the lethal side effect of the combination therapy since it happened at very early time after treatment. In this particular subgroup with older age, HMA combined with chemotherapy might even bring a worse effects on the final outcome. The reason for this finding probably due to, as certain studies had reported, a higher proportion of patients died of infection during the follow-up period for the combination group. It can be concluded that for patients with older age and fragile condition, higher CR did not necessary bring longer survival, so the treatment strategy should change from pursuing the short term CR rate to the improvement of the survival and quality of life for these patients in the future.

Our meta-analysis had recruited all the available related studies published for the past 10 years. Even though, there were still some limitations existed in this study. Significant heterogeneity was detected when data were pooled for statistical analysis, which might be explained by the heterogeneity in the real world. To solve this problem, we tried to use different statistical methods to diminish the possible error. On the other hand, the regimens in each group were not exactly consistent and the time for the publications spanned a long period when the methods of supportive care were quite different. Meanwhile, some publications had limited number of patients. Even though, our results showed that HMA combined with chemotherapy may not be the solution to improve the OS of HMA, especially for patients with older age. Higher intensity of the combined chemotherapy might favor the survival for patients with unfavorable cytogenetic risk and relatively younger age compared with low-intensity of the combined chemotherapy, but the benefit in survival disappeared for patients with older age. The combination of HMA and other regimens like BCL-2 inhibitors or histone deacetylase inhibitors may provide more efficient and less toxic options. One phase 1b study conducted by DiNardo et al.^31^ focused on venetoclax combined with either decitabine or azacitidine for AML patients, and a CR rate of 67% and two-year OS rate of 46% was observed. Other options like HMA combined with histone deacetylase inhibitor may also improve the efficacy, and as a result, improved the survival. New combination with HMA and target medicine with less adverse effects may come to the center of stage.

## Supplementary Material

Supplementary table.

## Figures and Tables

**Table 1:** Characteristics of selected studies

Therapy	Study	Publication type	HMA regimen	Combined chemotherapy regimen (induction therapy)	Combined chemotherapy regimen (Consolidation and maintenance therapy)	Median follow-up (month)
**Combination** **(high-intensity chemotherapy)**	Schlenk (2019)^17^	RCT	Azacitidine (100 mg/m^2^/day, day 1 to 5 every 28 days)	Idarubicin 12 mg/m^2^/day on days 6, 8, 10, etopsoside 100 mg/m^2^/day on days 6, 7, 8 every 28 days	High-dose cytarabine 3 g/m² bid on days 6, 7, 8, azacitidine 50 mg/m^2^/day, day 6 to10	56
	Müller- Tidow (2016)^19^	RCT	Azacitidine (75 mg/m^2^/day, day 1 to 5 every 28 days)	DA (cytarabine 100 mg/m^2^/day, day 6 to 12 and daunorubicin 60 mg/m^2^/day, day 8 to 10) every 28 days	CR patients: 2 courses of azacitidine 75 mg/m^2^ iv, day 1 to 5 and cytarabine 1 g/m^2^ iv over 3h, q12h, on days 6, 8, 10	Not reported
	Krug (2012)^20^	RCT	Azacitidine (75 or 37.5 mg/m^2^/ day, day 1 to 5 every 28 days)	DA (cytarabine 100 mg/m^2^/day, day 6 to 12 and daunorubicin 60 mg/m^2^/day, day 8 to 10) every 28 days	Responding patients: 2 courses of azacitidine 75 or 37.5 mg/m^2^/day, day 1 to 5, and cytarabine 1 g/ m^2^ bid, days 6, 8, and 10 every 28 days	20.5
	Scandura (2011)^18^	RCT	Decitabine (20 mg/m^2^/day, day 1 to 3/5/7 every 28 days)	DA (cytarabine 100 mg/m^2^/day, day 6 to 12 and daunorubicin 60 mg/m^2^/day, day 6 to 8) every 28 days	Not reported	32
**Combination** **(low-intensity chemotherapy)**	Huang (2018)^21^*	Retrospective cohort study	Decitabine (15 mg/m^2^/day, day 1 to 5 every 28 days)	CAG (cytarabine 10 mg/m^2^ q12h, day 3 to 9, aclarubicin 10 mg/day, day 3 to 6, and G-CSF 300 μg/d)	CR patients: Decitabine and CAG; cytarabine 2 g/m^2^ q12h for 3 days; for other patients: IA or FLAG	18
	Ye (2017)^22^	Retrospective cohort study	Decitabine (20 mg/m^2^/day, day 1 to 3 every 28 days)	IA (idarubicin 3 mg/m^2^/day for 4-6 days, cytarabine 10 mg/m^2^, q12h, for 14 days) or AA (aclacinomycin 10 mg/m^2^, q12h, for 6-8 days, cytarabine 10 mg/m^2^, q12h) following decitabine every 28 days	Not reported	10.9
	Li (2015)^23^	Retrospective cohort study	Decitabine (15 mg/m^2^/day, day 1 to 5 every 28 days)	CAG (cytarabine 10 mg/m^2^ q12h, day 3 to 9, aclarubicin 10 mg/day, day 3 to 6, and G-CSF 300 μg/d, day 0 to 9)	Nonresponding patients: cytarabine (100 mg/m^2^ q12h, 7 days, homoharringtonine 2 mg/ m^2^/day, 7 days, and daunorubicin 30 mg/ m^2^, 3 days)	Not reported
**HMA monotherapy**	Kanakasetty (2019)^32^	Retrospective cohort study	Azacitidine (100 mg/m^2^/day for 7 days every 28 days) or decitabine (20/ mg/m^2^ for 5 days every 28 days)	N/A	N/A	Not reported
	Ren (2019)^33^†	Retrospective cohort study	Decitabine (20 or 15 mg/m^2^/day, 5 days every 28 days)	N/A	N/A	31.4
	Fili (2018)^8^‡	Retrospective cohort study	Decitabine (20 mg/m^2^/day, 5 days every 28 days) as first-line or as salvage therapy	N/A	N/A	Not reported
	Almeida (2017)^34^‡	Retrospective cohort study	Azacitidine (75 mg/m^2^/day, 7 days every 28 days) as first-line or as salvage therapy	N/A	N/A	4.3
	Wu (2016)^35^	Retrospective cohort study	Decitabine (20 mg/m^2^/day, 5 days every 28 days)	N/A	N/A	Not reported
	Dombret (2015)^26^	RCT	Azacitidine (75 mg/m^2^/day, 7 days every 28 days)	N/A	N/A	24.4
	Gupta (2015)^36^	Retrospective cohort study	Azacitidine (75 mg/m^2^/day, 7 days every 28 days) or decitabine (20 mg/m^2^/day, 5 or 10 days every 28 days)	N/A	N/A	24.9
	Sadashiv (2014)^27^	RCT	Azacitidine (100 mg/m^2^/day, 7 days every 28 days)	N/A	N/A	Not reported
	van der Helm (2013)^25^	Retrospective cohort study	Azacitidine (100 mg/m^2^/day, 7 days every 28 days)	N/A	N/A	Not reported
	Al-Ali (2012)^37^‡	RCT	Azacitidine (75 mg/m^2^/day, 5 days every 28 days) as first-line or as salvage therapy	N/A	N/A	13
	Kantarjian (2012)^28^	RCT	Decitabine (20 mg/m^2^/day, 5 days every 28 days)	N/A	N/A	Not reported
	Lee (2011)^24^	Prospective cohort study	Decitabine (20 mg/m^2^/day, 5 days every 28 days)	N/A	N/A	15.9
	Cashen (2010)^29^	RCT	Decitabine (20 mg/m^2^/day, 5 days every 28 days)	N/A	N/A	Not reported
	Fenaux (2009)^9^	RCT	Azacitidine (75 mg/m^2^/day, 7 days every 28 days)	N/A	N/A	21.1

* Studies are recorded here in the name of first author and the publication year. †Only the subgroup with 20 mg/m2 of decitabine regimen was included in this meta-analysis according to the inclusion criteria. ‡Only patients treated with decitabine as first-line therapy was included in this meta-analysis according to the inclusion criteria. FLAG: fludarabine plus cytarabine and granulocyte colony stimulating factor (G-CSF); HMA: hypomethylating agents; RCT: randomized controlled trial; N/A: not applicable.

**Table 2:** Characteristics of patients participating in selected studies

Therapy	First Author	Disease type	No. of patients	Age (year), Median/ Average (Range/SD)	Male proportion/%	Cytogenetic risk: favorable(%)	Cytogenetic risk: intermediate(%)	Cytogenetic risk: unfavorable(%)	ECOG PS ≤ 1 (%)
**Combination** **Therapy** **(high-intensity chemotherapy)**	Schlenk	AML	168	62.7 (18-83)	56.5	0 (0%)	106 (63.1%)	62 (36.9%)	Not reported
	Müller-Tidow	AML	105	69.6±4.8	58.1	4 (4%)	59 (59%)	37 (37%)	87.2
	Krug	AML	12	68 (63-76)	50.0	0 (0%)	9 (75%)	3 (25%)	66.7
	Scandura	AML	30	54.5 (23-60)	53.3	0 (0%)	15 (53.6%)	13 (46.4%)	Not reported
**Combination** **Therapy** **(low-intensity chemotherapy)**	Huang	AML	52	64 (55-69)	63.5	9 (18%)	34 (68%)	7 (14%)	59.6
	Ye	MDS	40	55 (39-62)*	56.1	28 (75.7%)	7 (18.9%)	2 (5.4%)	Not reported
	Li	AML	91	68 (60-87)	61.5	1 (1.2%)	66 (80.5%)	15 (18.3%)	Not reported
**HMA monotherapy**	Kanakasetty	AML	58	64 (61-74)	67.2	14 (24.2%)	22 (37.9%)	22 (37.9%)	74.1
	Ren ‡	MDS	50	60.5 (51.5-69)*	64	31 (64.6%)	2 (4.2%)	15 (31.3%)	Not reported
	Fili§	AML	75	74 (65-84)	53.3	44 (80%)		11 (20%)	88.0
	Almeida§	AML	51	73 (46-89)	70.6	17 (33.3%)	23 (45.1%)	11 (21.6%)	Not reported
	Wu	MDS/AML	70	61 (20-82)	74.0	37 (52.9%)	16 (22.9%)	17 (24.3%)	Not reported
	Dombret	AML	241	75 (64-91)	57.7	0	155 (64.6%)	85 (35.4%)	77.2
	Gupta	AML	83	75.5 (60-92)	75.9	52 (62.7%)		31 (37.3%)	81.9
	Sadashiv	AML	15	74 (64-82)	60.0	0	9 (64.3%)	5 (35.7%)	Not reported
	van der Helm	AML	26	70 (60-81)	65	0	18 (69%)	8 (31%)	80.8
	Al-Ali§	AML	20	78 (32-84)	55	0	15 (75%)	5 (25%)	Not reported
	Kantarjian	AML	242	73 (64-89)	56.6	0	152 (63.6%)	87 (36.4%)	76.0
	Lee	MDS	101	65 (23-80)	67.3	65 (65.7%)	15 (15.1%)	19 (19.2%)	Not reported
	Cashen	AML	55	74 (61-87)	Not reported	0	29 (53.7%)	25 (46.3%)	81.8
	Fenaux	MDS/AML	179	69 (42-83)	73.7	83 (48.8%)	37 (21.8%)	50 (29.4%)	92.7

*The age of patients was presented as median (IQR) in this study. † The study enrolled both MDS and AML patients. ‡ Only the subgroup with 20 mg/m^2^ of decitabine regimen was included in this meta-analysis according to the inclusion criteria. § Only patients treated with decitabine as first-line therapy was included in this meta-analysis according to the inclusion criteria. AML: acute myeloid leukemia; ECOG PS: eastern cooperative oncology group performance state; MDS: myelodysplastic syndromes; SD: standard deviation

## Abbreviations

AA: aclacinomycin + cytarabine
AE: adverse events
AML: acute myeloid leukemia
CAG: cytarabine + aclarubicin + granulocyte colony stimulating factor
CR: complete response
CTCAE: Common Terminology Criteria for Adverse Events
DA: daunorubicin + cytarabine
ECOG PS: Eastern Cooperative Oncology Group Performance State
G-CSF: granulocyte colony stimulating factor
HAA: homoharringtonine + aclarubicin + cytarabine
HMA: hypomethylating agents
HSCT: hematopoietic cell transplantation
IA: idarubicin + cytarabine
MDS: myelodysplastic syndromes
ORR: overall response
OS: overall survival
PR: partial response
RCT: randomized controlled trial
t-MDS/AML: therapy-related MDS/AML
