# Supplementary material for: Comparison of Hypomethylator Monotherapy with Hypomethylator plus Chemotherapy for Intermediate/High-Risk MDS or AML: A Meta-Analysis
Source: J Cancer. 2020 Mar 4;11(10):2972–80. doi: 10.7150/jca.40614 (PMC7086269; doi:10.7150/jca.40614)
Supplement: Supplementary file 1 — Supplementary table. [file jcav11p2972s1.pdf]

Table S1 Reported data in each study and pooled estimates

**Study**	**CR rate (95%CI)**	**ORR rate (95%CI)**	**Two-year OS rate (95%CI)**	**CTCAE grade 3-4 infection rate (95%CI)**	**CTCAE grade 3-4 bleeding rate (95%CI)**	**CTCAE grade 3-4 cardiac disorder rate (95%CI)**	**CTCAE grade 3-4 bleeding rate (95%CI)**
Pooling model	Random	Random	Random	Random	Random	Random	Random
Schlenk	0.35 (0.27-0.42)	0.35 (0.27-0.42)	Excluded	0.66 (0.59-0.73)	0.06 (0.02-0.10)	0.12 (0.07-0.17)	0.17 (0.12-0.23)
Müller-Tidow	0.48 (0.38-0.58)	0.58 (0.48-0.68)	0.42 (0.32-0.52)	Not reported	Not reported	Not reported	Not reported
Krug	0.58 (0.30-0.86)	0.58 (0.30-0.86)	Unavailable*	Not reported	Not reported	0.08 (0.00-0.23)	Not reported
Scandura	0.57 (0.39-0.74)	0.90 (0.62-0.83)	Excluded	Not reported	Not reported	Not reported	0.30 (0.14-0.46)
Huang	0.60 (0.46-0.73)	0.73 (0.61-0.85)	0.44 (0.30-0.57)	0.17 (0.07-0.28)	Not reported	Not reported	Not reported
Ye	0.55 (0.40-0.70)	0.75 (0.62-0.88)	0.45 (0.30-0.60)	0.70 (0.56-0.84)	0.28 (0.14-0.41)	0.03 (0.00-0.07)	Not reported
Li	0.78 (0.69-0.87)	0.82 (0.74-0.91)	0.19 (0.11-0.27)	Not reported	Not reported	0.01 (0.00-0.03)	Not reported
I^2	87.8%	93.4%	84.2%	96.9%	88.5%	81.4%	51.3%
**Pooled estimate**	**0.55 (0.43-0.68)**	**0.67 (0.49-0.84)**	**0.37 (0.23-0.51)**	**0.51 (0.18-0.85)**	**0.16 (0.00-0.37)**	**0.05 (0.00-0.11)**	**0.21 (0.10-0.33)**
Kanakasetty	0.21 (0.10-31)	0.31 (0.19-43)	0.08 (0.01-0.15)	Not reported	Not reported	Not reported	Not reported
Ren	0.22 (0.11-0.33)	0.52 (0.38-0.66)	0.25 (0.13-0.37)	Not reported	Not reported	Not reported	Not reported
Fili	0.31 (0.20-0.42)	0.42 (0.30-0.54)	Unavailable*	Not reported	Not reported	Not reported	Not reported
Almeida	0.37 (0.24-0.51)	0.51 (0.37-0.65)	0.31 (0.18-0.44)	Not reported	Not reported	Not reported	Not reported
Wu	0.20 (0.11-0.29)	0.69 (0.58-0.79)	0.17 (0.09-0.26)	0.26 (0.16-0.36)	0.07 (0.01-0.13)	0	0
Dombret	0.34 (0.27-0.41)	0.38 (0.31-0.45)	0.16 (0.12-0.21)	Not reported	Not reported	Not reported	Not reported
Gupta	0.19 (0.11-0.28)	0.28 (0.18-0.37)	0.14 (0.07-0.22)	Not reported	Not reported	Not reported	Not reported
Sadashiv	0.20 (0.00-0.40)	0.33 (0.09-0.57)	0.17 (0.00-0.36)	0.07 (-0.06-0.19)	0.13 (-0.04-0.31)	Not reported	Not reported
van der Helm	0.35 (0.16-0.53)	0.42 (0.23-0.61)	0.35 (0.17-0.53)	0.35 (0.17-0.53)	Not reported	Not reported	Not reported

Al-Ali	0.05 (-0.02-0.12)	0.075 (-0.01-0.16)	Unavailable*	Not reported	Not reported	Not reported	Not reported
Kantarjian	0.16 (0.11-0.20)	0.30 (0.24-0.36)	0.11 (0.07-0.15)	0.26 (0.20-0.31)	Not reported	Not reported	Not reported
Lee	0.13 (0.06-0.19)	0.55 (0.46-0.65)	Excluded	Not reported	Not reported	Not reported	Not reported
Cashen	0.26 (0.14-0.37)	Not reported	0.12 (0.03-0.20)	0.24 (0.12-0.35)	Not reported	Not reported	Not reported
Fenaux	0.17 (0.11-0.22)	0.49 (0.42-0.57)	0.51 (0.43-0.58)	Not reported	Not reported	Not reported	Not reported
I^2	69.2%	78.1%	90.9%	55.5%	0.0%	Not applicable	Not applicable
**Pooled estimate**	**0.22 (0.18-0.26)**	**0.42 (0.35-0.48)**	**0.21 (0.13-0.29)**	**0.23 (0.16-0.30)**	**0.08 (0.02-0.13)**	**0**	**0**
P value	0.000	0.000	0.000	0.000	0.084	0.053	0.000

* The survival curve reported in the study did not reach a two-year end point.

CR: complete response; CTCAE: common terminology criteria for adverse events; ORR: overall response; OS: overall survival.
